# Management of metabolic syndrome by nutraceuticals prepared from chitosan and ferulic acid with or without beta-sitosterol and their nanoforms

**DOI:** 10.1038/s41598-023-38837-9

**Published:** 2023-07-27

**Authors:** Sahar Y. Al-Okbi, Ola Ali, A. S. Aly, D. Refaat, Reham S. H. Esmail, Hagar F. H. Elbakry

**Affiliations:** 1 Nutrition and Food Sciences Department, National Research Centre, Cairo, Egypt; 2 Biochemistry Department, Faculty of Pharmacy (Girls), Al Azhar University, Cairo, Egypt; 3 Preparatory and Finishing Department, National Research Centre, Cairo, Egypt; 4 Central Metallurgical R&D Institute (CMRDI), P.O box 87, Helwan, Egypt; 5 Instituto de Nanociencia y Materiales de Aragón, CSIC-Universidad de Zaragoza, 50018 Zaragoza, Spain; 6 Pathology Department, National Research Centre, Cairo, Egypt

**Keywords:** Biochemistry, Medical research

## Abstract

Dyslipidemia, steatohepatitis and insulin resistance are among the components of metabolic syndrome (MS). Nutraceuticals containing chitosan, beta-sitosterol and/or ferulic acid and their nanostructures could have a potential role for management of MS. The aim of the present study was to assess the efficacy of the aforementioned nutraceuticals in treatment of MS in rat and their interaction with atorvastatin, a hypolipidemic drug. The two nutraceuticals and their nanostructures were prepared and the nanostructures were assessed by transmission electron microscope and Fourier-Transform Infra-red Spectrometry. MS was induced in rats by feeding high fructose-high fat diet (HFFD). Different groups of rats fed HFFD and treated with the different nutraceuticals, atorvastatin and atorvastatin in combination with different nutraceuticals, control fed on balanced diet and control consumed HFFD without treatments were run. Plasma glucose, lipid profile, aminotransferases activity, total antioxidant capacity, malondialdehyde, urea, creatinine, insulin, high sensitivity C-reactive protein, and adiponectin were assessed along with calculation of insulin resistance. Liver fat and histopathology were investigated. All nutraceuticals in original and nanostructures showed beneficial effects in the treatment of MS, superiority was ascribed to nutraceuticals composed of chitosan and ferulic acid in both forms. A more promising treatment of MS belonged to atorvastatin administered with the different nutraceuticals.

## Introduction

Dietary–induced metabolic syndrome represents a global challenge with multiple directions. Wide consumption of fructose and saturated fat together with the sedentary lifestyle negatively impact human health and potentiate the progression of metabolic syndrome. Non-alcoholic fatty liver diseases (NAFLD) could be induced by consumption of these macronutrients and most probably lead to dyslipidemia^1^. The most serious health problem that could result from NAFLD is the development of cardiovascular diseases (CVDs)^2^. Dyslipidemia, visceral obesity, glucose intolerance, fatty liver, insulin resistance and hypertension are the components of metabolic syndrome^3^. These components predisposed individuals to type II diabetes and CVDs; inducing early mortality rate among people^4^.

Atorvastatin, a highly prescribed drug for management of dyslipidemia, has a low bioavailability^5^. To improve its effect physicians prescribe large atorvastatin dose but subsequently this may increases the drug side effects on liver and kidney. Moreover myopathy; one of the statin–associated side effects is shown to be due to the elevated levels of statin metabolites^6^. This side effect is very pronounced when a statin is taken with another type of cholesterol lowering drug, in particular fibrates. Statin is also prescribed to prevent CVDs in fatty liver patients^7^. Because of the side effects associated with the lipid-lowering drugs, the need for natural products with lipid-lowering potential and with minimal or no side effect is warranted. The bioactive ingredients purified from food called functional food ingredients or nutraceuticals are examples of such safe natural products. Nutraceuticals could decrease the absorption of fat and sugar from the intestinal tract as well as lowering plasma lipids and possess antioxidant and anti-inflammatory activities with the potential to reduce insulin resistance. Therefore, they could counteract dietary induced metabolic syndrome or might work as complementary to drug for treatment of such syndrome and its components^8^. Chitosan, a polymer of glucosamine, defined as a dietary fiber, which cannot be digested by digestive enzymes of humans is an example of such nutraceuticals. Chitosan is the only abundant polysaccharide derived from crustaceans, and its cationic characteristics are different from other dietary fibers. It exhibits a marked hypolipidemic, antioxidant and anti-inflammatory activities that would reduce the risk of CVDs and metabolic syndrome (MS) components^9^. Chitosan nanoparticles significantly reduced plasma viscosity and improved lipid profile of dietary induced dyslipidemia^10^. Nanochitosan is also used as a carrier for other active agents due to its high biocompatibility and safety^11^. Another nutraceuticals are phenolic compounds including ferulic acid that were shown to possess antihyperglycemic effect, anti-inflammatory and antioxidant effect^12,13^. β-sitosterol was shown to possess antioxidant, anti-inflammatory, and hypolipidemic effect^14^ and is another reported nutraceutical. In this context, it is hypothesized that administration of chitosan, ferulic acid and beta-sitosterol together could elicit beneficial effect towards MS with dyslipidemia and steatohepatitis components and that the preparation of such ingredients in nanostructure might have superior activity. On the other hand, drug-food interactions are well-known^15^. Among such interactions is the increased bioavailability and activity of atorvastatin during consumption of grapefruit; due to its content of phenolic acids as ferulic acid, caffeic acid, coumaric acid and sinapic acid^16^. Therefore, co-administration of nutraceuticals containing ferulic acid, beta-sitosterol and chitosan with atorvastatin may allow for the use of lower doses of statin; with subsequent reduction of its side effects.

The main objective of the present work was to study the remedial effect of nutraceuticals interventions towards MS with associated dyslipidemia and steatohepatitis in rat model. The tested nutraceuticals were those containing chitosan, beta-sitosterol and/or ferulic acid and their nanostructures. The aim included studying the possible interaction between atorvastatin and the studied nutraceuticals towards metabolic syndrome.

## Results

Nanoparticles of the nutraceuticals are typically prepared using techniques involving nanoprecipitation, and emulsion solvent evaporation.

### Particle size of nutraceuticals’ nanostructures

Transmission electron microscopy (TEM) showed that the particle size of both the Chitosan/ Ferulic acid (NN1) and the Chitosan/Ferulic acid/β-sitosterol (NN2) from nutraceuticals’ nanostructures were not exceeding 100 nm in at least one particle dimension (Fig. S1 in the supplement).

### Fourier-transform infra-red spectra (FT-IR) of nutraceuticals’ nanostructures

**Figure 1:**
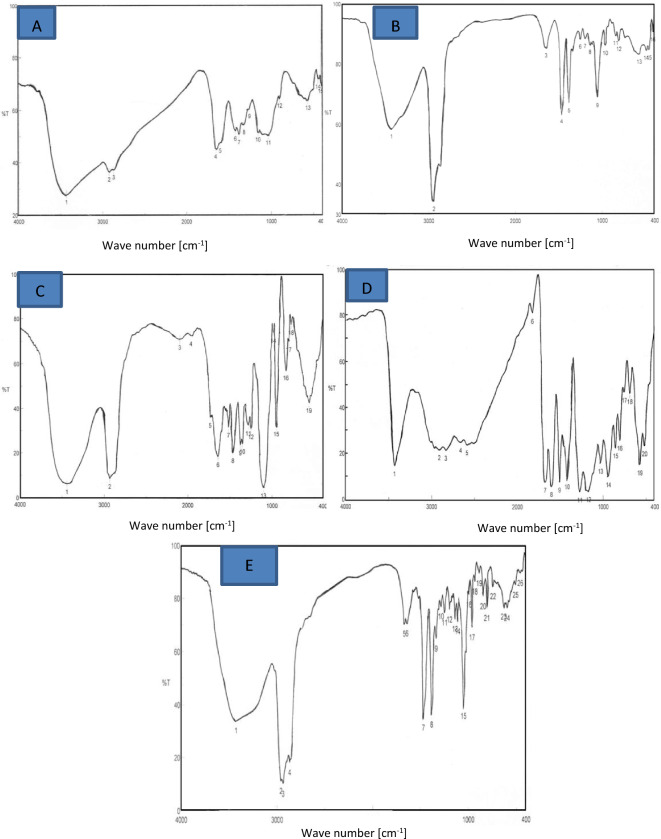
Fourier- transform infra-red spectra (FT-IR) of nano-nutraceuticals. (**A**) FT-IR spectra of chitosan, (**B**) FT-IR spectra of ferulic acid, (**C**) FT-IR spectra of β-sitosterol, (**D**) FT-IR spectra of chitosan/ferulic acid, (**E**) FT-IR spectra of chitosan/ferulic acid/β-sitosterol.

FT-IR was used to confirm the entrapping of the bioactive components (Ferulic acid and β-sitosterol) in the nutraceuticals’ nanostructures as shown in Fig. 1 and the Supplementary Tables S1–S5. Figure 1A,B,D and the Supplementary Tables S1, S2 and S4 proved that ferulic acid was successfully entrapped in the nanochitosan mesh (nanoscale chitosan-based material with a meshlike structure). This is because fifteen peaks were identified in chitosan spectra while 16 peaks were noticed in ferulic acid spectra; in case of chitosan/ ferulic acid nutraceutical nanostructure; the peaks numbers were 20 which were more than that of either chitosan or ferulic acid. Also it was noted that the position and intensity of the peaks were different in chitosan/ferulic acid nutraceutical compared to that of either chitosan or ferulic acid alone. On the other hand, Fig. 1C–E and the Supplementary Tables S3–S5 proved that both ferulic acid and beta-sitosterol were successfully entrapped in the nanochitosan mesh. The number of peaks of the nutraceutical nanostructure that contain chitosan, ferulic acid and beta-sitosterol were 26 compared to that of chitosan/ferulic acid nutraceutical nanostructure (20 peaks), in addition to difference in position and intensity.

### Results of the animal experiments

At the end of the first stage of the experiment, final body weight (FBW), body weight gain (BWG) and food efficiency ratio (FER) of rats fed on high fructose-high saturated fat diet (HFFD) for 2 months and exhibiting metabolic syndrome were significantly higher than that of the normal control (NC) group while food intake showed no change as demonstrated in the Supplementary Table S6.

In the second stage, the group fed on HFFD were divided into 10 sub-groups; metabolic syndrome control (MC), rats given atorvastatin drug (A), rats received nutraceutical 1 (N1), rats treated with nutraceutical nanoparticles1 (NN1), rats received nutraceutical 2 (N2), rats given nutraceutical nanoparticles 2 (NN2), rats taken atorvastatin drug with nutraceutical 1 (AN1), rats given atorvastatin drug with nutraceutical nanoparticles1 (ANN1), rats treated with atorvastatin drug with nutraceutical 2 (AN2), rats received atorvastatin drug with nutraceutical nanoparticles2 2(ANN2). It is to be noted that nutraceutical 1 consisted of chitosan with ferulic while nutraceutical 2 composed of chitosan + ferulic acid + β-sitosterol.

Data in Table 1 shows that at the beginning of the second stage; the metabolic syndrome control (MC) sub-group and all intervention sub-groups had a mean body weight (IBW in the second stage) of 255 g. Initial body weight of the normal control group in the second stage was significantly less than all the other sub-groups (207.5 g). After one month of interventions; final body weight of the normal control group was significantly the least (246.8 g); while FBW of the MC sub-group was significantly the highest (313.6 g) with FBW of all the intervention sub-groups lying in between them. Only, FBW of the sub-group that was given atorvastatin showed no change compared to the MC sub-group. BWG of the NC group was significantly low compared to that of the MC sub-group. BWG of all intervention sub-groups showed significant reduction compared to the MC sub-group. No changes in BWG values were noticed when the NC group was compared with all intervention sub-groups except for A and N2 that showed significantly higher values. BWG values of all treated sub-groups showed no changes compared to A except for AN1 and ANN1 that showed significant reduction. Total food intake of the MC sub-group was significantly higher than the NC group. All test sub-groups showed no change in food consumption compared to the NC group. Also, all test sub-groups demonstrated no changes in food intake values compared to the MC sub-group except for AN1 and ANN1 that exhibited significant reduction. Food efficiency ratio of the MC sub-group was significantly higher than that of the NC group. FER values of all test sub-groups were significantly lower than the MC sub-group except for A, N2 and NN2 that showed no changes.

**Table 1:** Nutritional parameters of different sub-groups at the end of the second stage of the experiment.

Groups	Parameters				
	IBW (g)	FBW (g)	BWG (g)	TFI (g)	FER
NC	207.5^a^ ± 4.4	246.8^a^ ± 3.57	39.3^a^ ± 1.46	378.3^a^ ± 13.04	0.104^a^ ± 0.005
MC	255.0^b^ ± 3.3	313.6^b^ ± 2.67	58.6^c^ ± 1.35	437.6^b^ ± 10.07	0.135^b^ ± 0.005
A	255.0^b^ ± 2.7	303.9^bc^ ± 1.96	48.9^b^ ± 1.09	418.4^ab^ ± 11.92	0.118^ab^ ± 0.005
N1	255.0^b^ ± 2.9	298.3^c^ ± 2.14	43.3^ab^ ± 1.35	408.9^ab^ ± 19.88	0.108^a^ ± 0.010
NN1	255.0^b^ ± 3.5	298.3^c^ ± 2.65	43.3^ab^ ± 1.86	390.3^ab^ ± 13.65	0.111^a^ ± 0.004
N2	255.0^b^ ± 3.7	304.0^c^ ± 2.77	49.0^b^ ± 2.27	403.8^ab^ ± 10.60	0.121^ab^ ± 0.004
NN2	255.0^b^ ± 3.3	301.0^c^ ± 2.98	46.0^ab^ ± 2.71	394.7^ab^ ± 11.20	0.116^ab^ ± 0.010
AN1	255.0^b^ ± 3.6	296.4^c^ ± 2.09	41.4^a^ ± 2.31	387.7^a^ ± 12.68	0.107^a^ ± 0.010
ANN1	255.0^b^ ± 3.8	296.9^c^ ± 2.96	41.9^a^ ± 2.38	377.2^a^ ± 8.54	0.112^a^ ± 0.010
AN2	255.0^b^ ± 2.5	298.3^c^ ± 2.35	43.3^ab^ ± 1.19	396.3^ab^ ± 7.99	0.110^a^ ± 0.010
ANN2	255.0^b^ ± 3.8	299.1^c^ ± 2.55	44.1^ab^ ± 1.39	395.7^ab^ ± 7.91	0.110^a^ ± 0.004

Values are expressed as means ± SE (n = 8). Significance was considered at p < 0.05 within columns where different letters indicate statistical significance.

*NC* control normal, *MC* metabolic syndrome control, *A* rats given atorvastatin drug, *N1* rats received nutraceutical 1, *NN1* Rats treated with nutraceutical 1nanostructure, *N2* rats received nutraceutical 2, *NN2* rats given nutraceutical 2 nanostructure, *AN1* rats taken atorvastatin drug with nutraceutical 1, *ANN1* rats given atorvastatin drug with nutraceutical 1 nanostructure, *AN2* rats treated with atorvastatin drug with nutraceutical 2, *ANN2* rats received atorvastatin drug with nutraceutical 2 nanostructure, *IBW* initial body weight, *FBW* final body weight, *BWG* body weight gain, *TFI* total food intake, *FER* food efficiency ratio.

Liver weight/body weight percent (Table 2) was of the highest value in the MC sub-group and was significantly higher than the NC group. Liver weight/ body weight % of different treated sub-groups showed significant reduction compared to the MC sub-group except for N2 and NN2 sub-groups that demonstrated no changes. Liver weight/body weight % showed no change when the nano-nutraceuticals (1 or 2) were compared with their corresponding nutraceuticals (1 or 2). When nutraceuticals either in normal or nanoform were given with atorvastatin they showed significant reduction in liver weight/ body weight % when compared with their corresponding nutraceuticals without atorvastatin. The lowest level of liver weight/ body weight % among treated sub-groups belonged to AN1 and ANN1. No treatment was statistically similar to the NC group.

**Table 2:** Liver weight/body weight (%) and liver TC and TG (mg/g) of the different sub-groups.

	Liver weight/BW (%)	Liver TC	Liver TG
NC	2.25^a^ ± 0.82	2.50^a^ ± 0.14	5.10^a^ ± 0.22
MC	3.74^b^ ± 0.15	7.35^b^ ± 0.21	14.60^b^ ± 0.62
A	3.19^c^ ± 0.04	4.95^c^ ± 0.09	9.29^c^ ± 0.26
N1	3.16^c^ ± 0.06	6.74^d^ ± 0.09	12.05^d^ ± 0.22
NN1	3.18^c^ ± 0.06	6.60^d^ ± 0.11	12.15^d^ ± 0.23
N2	3.58^b^ ± 0.10	6.89^bd^ ± 0.08	13.85^b^ ± 0.31
NN2	3.56^b^ ± 0.07	6.73^d^ ± 0.14	14.55^b^ ± 0.28
AN1	2.70^d^ ± 0.08	4.28^ef^ ± 0.37	7.66^e^ ± 0.19
ANN1	2.73^d^ ± 0.06	4.05^e^ ± 0.2	7.51^e^ ± 0.194
AN2	3.26^c^ ± 0.11	4.71^cf^ ± 0.09	9.21^c^ ± 0.30
ANN2	3.16^c^ ± 0.04	5.10^c^ ± 0.22	9.59^c^ ± 0.32

Values are expressed as means ± SE (n = 8). Significance was considered at p < 0.05 within columns where different letters indicate statistical significance.

*NC* control normal, *MC* metabolic syndrome control, *A* rats given atorvastatin drug, *N1* rats received nutraceutical 1, *NN1* rats treated with nutraceutical 1nanostructure, *N2* rats received nutraceutical 2, *NN2* rats given nutraceutical 2 nanostructure, *AN1* rats taken atorvastatin drug with nutraceutical 1, *ANN1* rats given atorvastatin drug with nutraceutical 1 nanostructure, *AN2* rats treated with atorvastatin drug with nutraceutical 2, *ANN2* rats received atorvastatin drug with nutraceutical 2 nanostructure, *BW* body weight, *TC* total cholesterol, *TG* triglycerides.

Liver total cholesterol (TC) in Table 2 was significantly higher in the MC sub-group than all other groups. Significant reduction in liver TC from that of the MC sub-group was noticed in all intervention sub-groups except for N2 that showed no change. No changes were noticed in liver TC when the sub-groups treated with N1or N2 were compared with that given the corresponding nano-nutraceuticals. Significant reduction was noticed in liver TC when Atorvastatin was given with any of the nutraceuticals either in original or nanoform compared to MC sub-group. No treatment could reduce liver TC to the level of NC group. Liver triglycerides (TG) of the MC sub-group were significantly higher than that of NC group (Table 2). All tested sub-groups showed significant reduction in liver TG compared to the MC sub-group except for N2 and NN2 that showed no changes. When Atorvastatin was administered with either nutraceuticals or their nanoforms there were significant reductions in liver TG compared to the sub-groups given only the corresponding nutraceuticals or the nano-forms. The liver TG content from the highest to the lowest was as follows: MC, NN2, N2, NN1, N1, ANN2, A, AN2, AN1, ANN1 and NC. Although the different treatments produced reduction in liver TG, their values are still higher than the NC group.

Plasma lipid profile of all sub-groups is shown in Table 3. The NC group showed a significantly low TC level among all other sub-groups; while the MC sub-group showed a significantly higher TC levels among all the sub-groups. The best improvement in TC was in case of AN1 and ANN1administration. TC of AN1 and ANN1 were significantly low compared to A, N1 and NN1 groups. TC of the sub-group A was significantly lower than MC, N1, NN1, N2 and NN2. No change in TC was noticed between N1 and NN1 while that of NN2 was significantly lower than N2. AN2 and ANN2 showed significant reductions in TC compared to N2, NN2 and A. Low density lipoprotein cholesterol (LDL-C) showed significant increase in the MC sub-group compared to the NC group, while different treatments demonstrated significant reductions compared to the MC sub-group. In respect to high density lipoprotein-cholesterol (HDL-C) levels; a significant decrease was noticed in the MC sub-group compared to NC group. Only the groups treated with atorvastatin alone or in conjunction with the different nutraceuticals (normal or nano-form) produced significant increase in HDL-C which could reach the normal level in the NC group. Atherogenic index (TC/HDL-C) was significantly high in the MC sub-group compared to the NC group. All treated sub-groups showed significant reduction in TC/HDL-C compared to the MC sub-group which could not reach the normal level (in the NC group). Comparing the sub-groups given the nutraceuticals with those administered the corresponding nano-nutraceuticals showed no changes in TC/HDL-C. The MS rats that given the different nutraceuticals (original and nanoform) in conjunction with atorvastatin showed significant reduction in TC/HDL-C compared to those given the corresponding nutraceuticals alone and compared to A group. Non HDL-C of the MC sub-group was significantly higher than the NC group. All treated groups showed significant reduction in non HDL-C compared to the MC sub-group. The pattern of non HDL-C in different treated groups is similar to that of TC/HDL-C. The changes in plasma LDL-C and TG of different groups were almost similar to that of non HDL-C.

**Table 3:** Plasma lipid profile (mg/dL) and atherogenic indices (TC/HDL-C and Non HDL-C) of the different sub-groups.

Group	TC	HDL-C	TC/HDL-C	Non HDL-C	LDL-C	TG
NC	84.38^a^ ± 1.74	46.75^a^ ± 1.82	1.83^a^ ± 0.1	37.63^a^ ± 2.83	19.8 ^a^ ± 2.9	89.0^a^ ± 2.10
MC	307.50^b^ ± 11.40	39.40^b^ ± 1.27	7.84^b^ ± 0.3	268.1^b^ ± 10.9	236.4 ^b^ ± 9.9	158.5^b^ ± 6.50
A	197.80^c^ ± 6.14	43.25^a^ ± 1.31	4.58^c^ ± 0.2	154.1^c^ ± 5.7	131.9 ^c^ ± 5.8	110.7^c^ ± 4.60
N1	259.80^d^ ± 6.00	38.40^b^ ± 1.29	6.84^d^ ± 0.3	221.4^d^ ± 6.6	197.4 ^d^ ± 5.8	120.1^d^ ± 5.04
NN1	267.50^d^ ± 6.53	40.50^b^ ± 1.05	6.63^d^ ± 0.2	227^d^ ± 6.5	201.8 ^d^ ± 6.5	125.6^d^ ± 2.50
N2	277.50^e^ ± 5.05	40.40^b^ ± 1.28	6.93^d^ ± 0.3	237.1^d^ ± 5.4	210.5 ^d^ ± 5.4	133.2^df^ ± 1.98
NN2	275.30^d^ ± 6.20	40.60^b^ ± 0.5	6.8^d^ ± 0.2	234.6^d^ ± 6.4	207.1^d^ ± 6.2	137.4^f^ ± 2.00
AN1	122.00^f^ ± 2.58	47.25^a^ ± 1.63	2.61^e^ ± 0.1	74.75^e^ ± 2.99	53.9^e^ ± 3.4	104.1^ce^ ± 3.50
ANN1	123.60^f^ ± 2.80	45.25^a^ ± 0.77	2.7^eg^ ± 0.1	78.38^e^ ± 3.54	58.6 ^e^ ± 3.7	98.75^ae^ ± 3.04
AN2	148.50^g^ ± 3.60	46.75^a^ ± 1.39	3.2^fg^ ± 0.1	101.8^f^ ± 3.4	80.3^f^ ± 3.8	107.2^ce^ ± 4.30
ANN2	155.60^g^ ± 3.10	46.00^a^ ± 1.16	3.4^f^ ± 0.1	109.6^f^ ± 3.6	87.7^f^ ± 3.1	109.8^ce^ ± 3.80

Values are expressed as means ± SE (n = 8). Significance was considered at p < 0.05 within columns where different letters indicate statistical significance.

.*NC* control normal, *MC* metabolic syndrome control, *A* rats given atorvastatin drug, *N1* rats received nutraceutical 1, *NN1* rats treated with nutraceutical 1nanostructure, *N2* rats received nutraceutical 2, *NN2* rats given nutraceutical 2 nanostructure, *AN1* rats taken atorvastatin drug with nutraceutical 1, *ANN1* rats given atorvastatin drug with nutraceutical 1 nanostructure, *AN2* rats treated with atorvastatin drug with nutraceutical 2, *ANN2* rats received atorvastatin drug with nutraceutical 2 nanostructure, *TC* total cholesterol, *TG* triglycerides, *HDL-C* high density lipoprotein-cholesterol, *LDL-C* low density lipoprotein-cholesterol.

Table 4 showed that there were statistical significant increases in alanine transamiase (ALT) and aspartate transaminase (AST) activities and creatinine and urea when the MC sub-group was compared with the NC group. The sub-group taken atorvastatin drug showed statistical improvement in these biomarkers relative to the MC sub-group. Also all the intervention sub-groups showed more improvements in these biomarkers than atorvastatin drug when used alone but with different degrees.

**Table 4:** Plasma ALT and AST activities, biomarkers of kidney functions (creatinine and urea), plasma glucose, insulin, and insulin resistance (IR) of different sub-groups.

Group	ALT (IU/L)	AST (IU/L)	Creatinine (mg/dL)	Urea (mg/dL)	Glucose (mg/dl)	Insulin (µIU/ml)	IR
NC	33.9^a^ ± 1.37	75.2^a^ ± 1.57	0.631^a^ ± 0.010	31.9^a^ ± 0.84	74.8 ^a^ ± 1.37	14.5 ^a^ ± 0.7	2.6 ^a^ ± 0.11
MC	63.9^b^ ± 1.83	144.5^b^ ± 2.47	0.800^b^ ± 0.013	48.5^b^ ± 1.42	119.2^b^ ± 3.80	20.7^b^ ± 0.6	6.1^b^ ± 0.26
A	51.4^c^ ± 0.85	105.3^c^ ± 2.62	0.660^ac^ ± 0.015	39.0^c^ ± 0.89	85.3^c^ ± 2.22	19.3^b^ ± 0.6	4.1^c^ ± 0.17
N1	42.3^d^ ± 0.86	107.3^cd^ ± 3.01	0.664^ac^ ± 0.020	39.8^c^ ± 1.06	80.1^d^ ± 0.98	19.8^b^ ± 0.6	3.9^c^ ± 0.13
NN1	42.5^d^ ± 0.93	103.8^c^ ± 1.57	0.670^ac^ ± 0.014	39.9^c^ ± 0.49	78.5^d^ ± 1.07	19.8^b^ ± 0.5	3.8^c^ ± 0.12
N2	47.5^e^ ± 0.75	121.9f. ± 1.66	0.713^d^ ± 0.020	45.5^d^ ± 0.44	84.2^c^ ± 1.10	19.8^b^ ± 0.6	4.1^c^ ± 0.13
NN2	46.1^e^ ± 1.11	121.2f. ± 2.54	0.708^d^ ± 0.080	43.9^d^ ± 0.33	85.3^c^ ± 0.79	20.0^b^ ± 0.4	4.2^c^ ± 0.09
AN1	40.8^d^ ± 0.52	88.7^e^ ± 1.89	0.632^a^ ± 0.010	34.6^e^ ± 0.81	74.8^a^ ± 0.92	16.7^c^ ± 0.3	3.1^d^ ± 0.08
ANN1	42.2^d^ ± 1.18	89.7^e^ ± 0.91	0.629^a^ ± 0.010	34.2^ae^ ± 1.02	73.9^a^ ± 1.04	16.9^c^ ± 0.3	3.1^d^ ± 0.08
AN2	46^e^ ± 1.26	108.6^cd^ ± 1.86	0.690^cd^ ± 0.010	39.1^c^ ± 0.86	83.0^c^ ± 1.10	19.0^b^ ± 0.4	3.8^c^ ± 0.11
ANN2	47.0^e^ ± 0.90	112.6^d^ ± 1.04	0.670^cd^ ± 0.010	40.5^c^ ± 0.85	82.1^c^ ± 1.37	18.8^b^ ± 0.7	3.9^c^ ± 0.15

Values are expressed as means ± SE (n = 8). Significance was considered at p < 0.05 within columns where different letters indicate statistical significance.

*NC* control normal, *MC* metabolic syndrome control, *A* rats given atorvastatin drug, *N1* rats received nutraceutical 1, *NN1* rats treated with nutraceutical 1nanostructure, *N2* rats received nutraceutical 2, *NN2* rats given nutraceutical 2 nanostructure, *AN1* rats taken atorvastatin drug with nutraceutical 1, *ANN1* rats given atorvastatin drug with nutraceutical 1 nanostructure, *AN2* rats treated with atorvastatin drug with nutraceutical 2, *ANN2* rats received atorvastatin drug with nutraceutical 2 nanostructure, *ALT* alanine transaminase, *AST* aspartate transaminase, *IR* insulin resistance.

Table 4 shows plasma glucose, insulin and insulin resistance of the NC group and MC sub-groups with or without interventions. The MC sub-group demonstrated significant high plasma glucose level compared to the NC group. All treated MS rats showed significant reduction of plasma glucose compared to the MC sub-group which only matched normal levels in case of AN1 and ANN1 groups. The NC group showed the significantly lowest plasma insulin levels. No changes in plasma insulin was shown among MC, A, N1, NN1, N2, NN2, AN2 and ANN2. Only AN1 and ANN1 showed marked reduction in plasma insulin levels relative to the MC group. Insulin resistance showed significant increase in the MC sub-group compared to the NC group. Insulin resistance was significantly improved in all treatments compared to the MC sub-group. Insulin resistance of all treated groups was still significantly lower than NC group. The best improvement in insulin resistance belonged to AN1 and ANN1 groups.

Table 5 shows plasma total antioxidant capacity (TAC), malondialdehyde (MDA), adiponectin and high sensitivity-C reactive protein (hs-CRP) of different sub-groups. Plasma total antioxidant capacity of MC sub-group was significantly lower than that of the NC group. Significant improvement in TAC was noticed on different treatments compared to the MC group. No changes in TAC values were noticed when MS rats treated with nano-nutraceuticals were compared with those treated with the corresponding original nutraceuticals. Sub-groups treated with atorvastatin in conjunction with nutraceuticals (nano-form and original) showed significant increase in TAC compared to those given only the corresponding nutraceuticals. AN1 and ANN1 sub-groups showed significant elevation of TAC compared to A sub-group. Malondialdehyde was significantly high in the MC sub-group compared to the NC group. Different treatments improved MDA level compared to the MC group. The best improvement in MDA level was related to AN1 and ANN1 groups.

**Table 5:** Plasma TAC, MDA, adiponectin and hs-CRP of different experimental sub-groups.

Group	TAC (mM/L)	MDA (nmole/ml)	Adiponectin (µg/ml)	hs-CRP (ng/L)
NC	6.68^a^ ± 0.2	5.27^a^ ± 0.20	7.04^a^ ± 0.11	107.9^a^ ± 3.9
MC	3.49^b^ ± 0.2	8.30^b^ ± 0.07	3.36^b^ ± 0.11	227.6^b^ ± 24.2
A	4.49^c^ ± 0.1	6.30^cd^ ± 0.12	5.99^c^ ± 0.11	120.9^acd^ ± 1.8
N1	4.54^c^ ± 0.1	6.80^ef^ ± 0.12	4.94^d^ ± 0.08	129.4^acd^ ± 2.2
NN1	4.51^c^ ± 0.1	6.60^ce^ ± 0.11	4.98^d^ ± 0.08	125.5^acd^ ± 2.0
N2	3.75^b^ ± 0.2	7.20^g^ ± 0.11	4.40^e^ ± 0.13	129.9^cd^ ± 2.3
NN2	3.85^b^ ± 0.1	6.90^fg^ ± 0.07	4.70^de^ ± 0.11	132.9^d^ ± 1.8
AN1	5.40^d^ ± 0.2	5.70^h^ ± 0.11	6.68^f^ ± 0.10	111.1^ac^ ± 3.2
ANN1	5.20^d^ ± 0.1	5.80^h^ ± 0.11	6.56^f^ ± 0.12	113.1^acd^ ± 2.2
AN2	4.49^c^ ± 0.1	6.20^d^ ± 0.11	5.90^c^ ± 0.15	123.9^acd^ ± 3.1
ANN2	4.40^c^ ± 0.1	6.00^d^ ± 0.07	6.20^c^ ± 0.13	123.3^acd^ ± 1.6

Values are expressed as means ± SE (n = 8). Significance was considered at p < 0.05 within columns where different letters indicate statistical significance.

*NC* control normal, *MC* metabolic syndrome control, *A* rats given atorvastatin drug, *N1* rats received nutraceutical 1, *NN1* rats treated with nutraceutical 1nanostructure, *N2* rats received nutraceutical 2, *NN2* rats given nutraceutical 2 nanostructure, *AN1* rats taken atorvastatin drug with nutraceutical 1, *ANN1* rats given atorvastatin drug with nutraceutical 1 nanostructure, *AN2* rats treated with atorvastatin drug with nutraceutical 2, *ANN2* rats received atorvastatin drug with nutraceutical 2 nanostructure, *TAC* total antioxidant capacity, *MDA* malondialdehyde, *hs-CRP* high sensitivity C-reactive protein.

Considering adiponectin; the NC group showed the statistically highest adiponectin value while the MC sub-group showed the statistically lowest adiponectin levels. All treated rats showed significant improvement in adiponectin levels compared to the MC sub-group. AN1 and ANN1showed the highest statistically significant levels in adiponectin relative to the MC sub-group followed by ANN2, A and AN2. In respect to hs-CRP levels; there was a statistically significant increase in the MC sub-group compared to the NC group. All treated MS sub-groups showed significant reduction in hs-CRP compared to the MC sub-group which matched the control level except for N2 and NN2 sub-groups.

The correlation study (Supplementary Table S7) showed plasma glucose and lipids (except HDL-C) were positively correlated with all the determined biochemical parameters except for TAC and adiponectin. Plasma glucose and lipids were negatively correlated with TAC and adiponectin. Also biomarkers of liver and kidney functions (ALT, AST, creatinine and urea) were positively correlated with all the determined biochemical parameters except for HDL-C, TAC and adiponectin. Plasma ALT, AST, urea and creatinine were negatively correlated with HDL-C, TAC and adiponectin. TAC was significantly and negatively correlated with MDA. Insulin was positively correlated with all parameters except TAC, MDA, adiponectin and hs-CRP. Insulin is negatively correlated with TAC, MDA, adiponectin and hs-CRP. Adiponectin was negatively correlated with all biochemical parameters except for HDL-C, MDA and TAC. Adiponectin was positively correlated with HDL-C, MDA and TAC. hs-CRP was positively correlated with all the determined biochemical parameters except for HDL-C, TAC and adiponectin. hs-CRP was negatively correlated with HDL-C, TAC and adiponectin.

### Histopathological results

**Figure 2:**
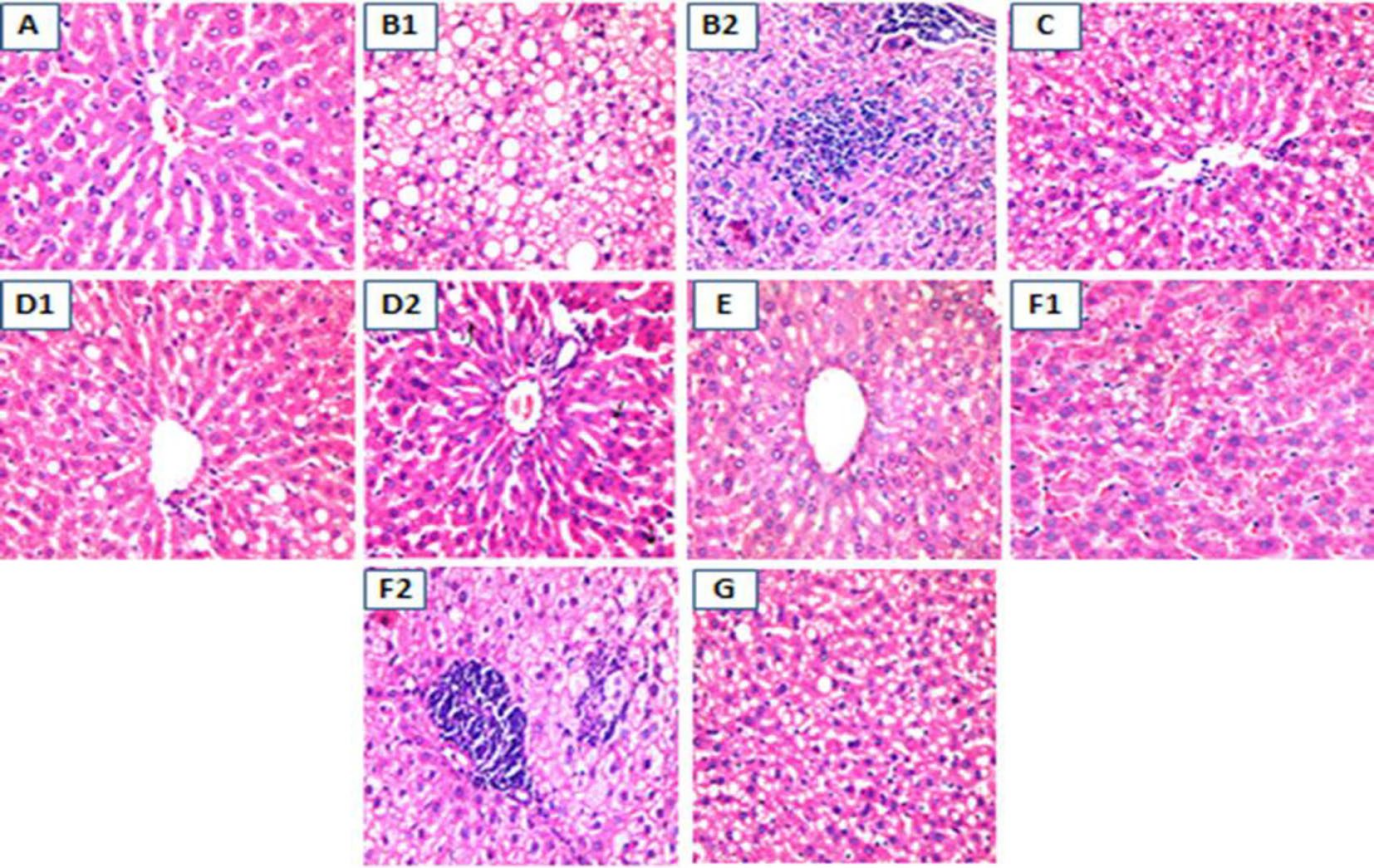
Histopathological changes of liver of different experimental sub-groups. (**A**) Liver histopathology of rat of normal control group showed normal appearance(H &E, X 400), (**B1**) liver histopathology of MC sub-group showed severe steatosis (H&E, X400), (**B2**) liver histopathology of MC sub-group showed inflammatory cells collections of variable intensity (H&E, X400), (**C**) liver histopathology of atorvastatin treated sub-group showed relative decrease in hepatic steatosis intensity and surface area (H&E, X200), (**D1**) Liver histopathology of atorvastatin + nutraceutical 1 treated group: Liver showed much decrease in steatosis intensity and surface area than atorvastatin alone treated sub-group (H&E, X400), (**D2**) Liver histopathology of atorvastatin + nutraceutical 1 treated group: Liver showed excellent response in some rats with occasional fatty vacuoles (H&E, X400), (**E**) liver histopathology of rats treated with atorvastatin + nutraceutical 2, liver tissue with steatotic vacuoles in about 30% surface area (H&E, X400), (**F1**) liver histopathology of rats taken nutraceutical 1 only, some rats showed foamy vacuolated cytoplasm of the hepatocytes and some inflammation. (**F2**) Liver histopathology of rats taken nutraceutical 1 only, the hepatocytes of the rats had foamy cytoplasm with focal inflammatory cells but no steatosis (H&E, X400), (**G**) liver histopathology of rats taken nutraceutical 2 only, the hepatic steatosis was about 50% of the examined area.

The histopathology of liver is demonstrated in Fig. 2. The subgroups that were histopathologically examined were the NC group, the MC, A, AN1, AN2, N1 and N2 sub-groups. Liver of the NC rats (Fig. 2A) had a normal appearance. On the other hand liver tissue of the MC sub-group (Fig. 2B1,B2) showed severe steatosis with variable sized fatty vacuoles (macro and micro-vesicles) in about 70% surface area, aggregates of inflammatory cells are also seen.

Liver of rats treated with Atorvastatin (Fig. 2C) demonstrated relatively lower intensity and surface area of steatosis (fatty vacuoles); about 40–50% of surface area, than its rates in the MC sub-group. Liver sections of rats treated with both atorvastatin and nutraceutical 1 (Fig. 2D1,D2) showed mild to moderate steatosis; of decreased intensity and surface area than those of rats treated with atorvastatin only (about 10- 20% of surface area). Also some rats showed excellent response with minimal residual steatosis. Liver sections of rats treated with atorvastatin with the nutraceutical 2 (Fig. 2E) revealed liver tissue showing fatty changes in about 30%of the surface area. Rats that were given nutraceutical 1(Fig. 2F1,F2) only showed fatty changes in the hepatic tissue of variable degrees reaching up to 30% and down to 15 or 10% (H&E, X 400). Some rats showed only foamy vacuolated cytoplasm and some inflammation of the hepatocytes vesicles, without steatosis. In rats that were given nutraceutical 2 (Fig. 2G) only the hepatic steatosis was about 50% of the area. However the effect among the rats in all test sub-groups generally was heterogeneous with some of them showed good to excellent response, while the others showed limited response. According to percent of fat deposition in liver, the arrangement of groups could be as the following (from high fat deposition to the lower deposition):

$${\text{MC}}\, > \,{\text{N2}}\, > \,{\text{A}}\, > \,{\text{AN2}}\, > \,{\text{N1}}\, > \,{\text{AN1}}.$$

## Discussion

Metabolic syndrome composes of different metabolic changes comprises central obesity, dyslipidemia, hyperglycemia and hypertension with fatty liver as hepatic component. Such changes could lead to CVDs and type-2 diabetes^17^.

In the present work two nutraceuticals were prepared; the first was made from chitosan and ferulic acid and the second consisted from chitosan, ferulic acid and beta-sitosterol. Two extra nutraceuticals nanoparticles were prepared from these two nutraceuticals and were examined by infrared and electron microscopy that verified both inclusion of the different compounds and their occurrence in nanoform. The four nutraceuticals were evaluated in metabolic syndrome model of rats to study their potential therapeutic effect. A hypolipidemic drug (atrovastatin) was used with or without the different nutraceuticals to study the possible drug-nutraceuticals interaction as therapeutic agents.

In the present study, feeding HFFD was able to induce metabolic syndrome indicated by elevated oxidative stress, inflammation, fatty liver, elevated plasma glucose, insulin resistance, dyslipidemia, elevated cardiovascular risk and increased body weight. These changes were reflected in the increased non HDL-C and TC/HDL-C, elevated plasma MDA and reduction of TAC with increased liver fat and liver weight. Also, inflammation was mirrored in reduced adioponectin and elevated hs-CRP. Liver histopathology showed high fat accumulation along with inflammation in the MC sub-group which confirmed the biochemical results. It could be noticed in the current work that MS was associated with both liver and kidney dysfunction designated by elevated transaminases’ activities and urea and creatinine levels.

Intake of high fructose diet was reported to induce excessive generation of reactive oxygen species and liver failure with development of metabolic syndrome that characterized by obesity, insulin resistance, hypercholesterolemia, hypertriglyceridemia and hepatic steatosis^18^. Fructose is a highly lipogenic sugar that stimulates de novo lipogenesis with profound metabolic effects in the liver^19^. On the other hand, animal studies showed that saturated fat-rich diet induced steatosis, promotes endoplasmic reticulum stress and hepatocyte injury^20^ with increased insulin resistance and dyslipidemia^21^. Bile salts; in the present experimental diet could enhance cholesterol absorption that doubtless induced hypercholesterolemia. So the presence of both saturated fat and fructose in the diet in the present study together with cholesterol and bile salts could have a synergistic effect in induction of MS and steatohepatitis.

Deposition of liver fat lead to increased secretion of VLDL-C, hypertriglyceridemia, elevated LDL-C and decreased HDL-C^22^ as noticed in the MC sub-group in the present study showing the CVD risk due to fatty liver that further emphasized by the elevated TC/HDL-C. Hypertriglyceridemia noticed in the present study may result from feeding high fructose diet and the subsequent liver triglycerides synthesis. Fructose is more rapidly metabolized than glucose as its metabolism bypass fructose1-6-bisphosphate step; one of the rate limiting steps in glycolysis. Hepatic metabolism of fructose gives trioses by the action of aldolase B. These trioses are the backbone of TG. Fructose can inhibit beta oxidation of fatty acids, and subsequently leads to hepatic TG accumulation^23^. Endothelial dysfunctions could result from the direct effects of the hypertriglyceridemia on the arterial walls^24^.

Cooney et al*.*^25^ reported that there is a strong inverse relation between HDL-C concentration and CVDs. Thus the decrease in HDL-C and the increase in TC and non HDL-cholesterol are greatly acceptable as risk factors for CVDs^26^. Studies on HDL-C prove its antioxidant and antiinflammatory role inside the body. In addition to the role of HDL in transport of lipids from body tissues to the liver (reverse cholesterol transport); HDL also contains paraoxonase enzyme with an antioxidant properties^27^. The anti-inflammatory effect of HDL can reduce vascular inflammation^28^.

There was a notable improvement in both dyslipidemia and TC/HDL-C on treatment with atorvastatin and nutraceuticals with or without atorvastatin. Generally speaking; the improvement was more pronounced when nutraceuticals was given with atorvastatin. Nutraceutical 1 was more efficient than 2 and nutraceuticals nanoparticles were more promising than the original nutraceuticals concerning the majority of lipid parameters. So generally the arrangement could be ANN1 > AN1 > ANN2 > AN2 > A > NN1 > N1 > NN2 > N2 with some little variations concerning the individual lipid parameters. An exception is that nutraceuticals either as nanostructure or original form (N1, NN1, N2 and NN2) could not elicit any sensible effect concerning HDL-C.

Liver TG and cholesterol were efficiently reduced on different treatments. AN1 and ANN1 were superior in reducing both liver TG and cholesterol. As hepatocytes are capable for regeneration; nutraceuticals could improve both steatosis and dyslipidemia during the present study.

Although combination of chitosan with ferulic acid and beta-sitosterol has a significant improvement of dyslipidemia however the effect was lower than chitosan with ferulic acid which could be due to that beta-sitosterol might tightly binds to chitosan as reported by Vahouny et al*.*^29^. This binding could slightly reduce the action of chitosan in hindering cholesterol absorption from the intestine which in turns leads to slight reduction of the efficacy of the nutraceutical in treating dyslipidemia as shown from the results. Chitosan is not only a well-known matrix for nanoproducts^30^ but also a hypolipidemic agent. The hypolipidemic effect of chitosan might be attributed to binding to dietary lipids in the intestine thereby reduces lipid absorption resulting in reduction of blood cholesterol in both animals and human^31,32^. The strong positive charge carried by the chitosan makes it binds well to lipids (negatively charged). Topping^33^ reported that chitosan fermentation by microbiota in the large intestine results in short chain fatty acids that can enter the circulation and suppress the *in-vivo* cholesterol synthesis. However Bokura and Kobayashi^32^ documented that the effect of chitosan on decreasing total and LDL-C is mild.

Phenolic group in ferulic acid can improve distribution of cholesterol in the lipoproteins mainly due to the inhibition of cholesterol ester transfer protein activity^34^. Ferulic acid also was reported to improve blood viscosity^35^ thereby it could have therapeutic roles towards CVD. Beta-sitosterol hypolipidemic effect might be attributed to its resemblance to cholesterol in the chemical structure. This in turns reduces the intestinal cholesterol absorption by competing of the beta-sitosterol with the dietary cholesterol for the absorption site. A study by Field et al*.*^36^ reported that beta sitosterol can decrease cholesterol synthesis in CaCo-2 cells by reducing HMG CoA reductase gene and protein products. Significant reduction in the activity of hepatic HMG CoA reductase in sitosterolemic patients is also documented^37^.

The mechanism of atorvastatin as hypocholesterolemic drug is attributed to prevention of cholesterol synthesis by inhibiting HMG-CoA. The efficacy of atorvastatin is affected by its metabolism in the liver via cytochrome P 450 enzyme^38^ which could be affected due to induction of fatty liver in the present study. High dose of atorvastatin (10 mg/kg rat body weight/day) is used in the present study because atorvastatin is of compromised low oral bioavailability and is also highly metabolized in the rat^39^. Atorvastatin was reported to reduce LDL–C by 25–60%^40^.

Plasma MDA and total antioxidant activity in the present work were improved on different treatments. The sequence of improvement of MDA from the highest to the lowest was AN1 > ANN1 > ANN2 > AN2 > A > NN1 > N1 > NN2 > N2 while that of TAC was AN1 > ANN1 > N1 > NN1 > A = AN2 > ANN2. NN2 and N2 administration showed no improvement in TAC however they could reduce lipid peroxidation. Significant decrease in MDA with the elevation of TAC in the intervention groups suggested that nutraceuticals exhibit either direct antioxidant effect or have ability to scavenge reactive oxygen species. Inflammation biomarkers represented by adiponectin and hs-CRP were significantly improved on different treatments; the superiority belongs to both AN1 and ANN1 while the inferiority belongs to N2 and NN2. Chitosan antioxidant activity is attributed to the reaction of unstable free radicals with amino and hydroxyl groups on the pyranose ring of chitosan, which form stable radicals^41^. Chitosan was also reported to have an anti-inflammatory effect^42^. Regarding ferulic acid; the hydroxy phenyl moeity is responsible for its antioxidant effect. The phenoxy radical of ferulic acid is stable due to resonance. The carboxylic acid moeity of ferulic acid acts as an anchor by which ferulic acid binds to the lipids, protecting them against lipid peroxidation. Thus the stable resonance structure of the phenoxy radical is necessary to stop the propagation of any chain reaction initiated by free radicals, making ferulic acid especially able to scavenge and stop free radical chain reactions^43^. Ferulic acid ethyl esters were reported to have anti-inflammatory activity^44^. Ferulic acid is a phenolic compound and generally phenolic compounds were reported to reduce NAFLD, cardiovascular disease and diabetes^45^. These therapeutic effects of phenolic compounds might be attributed to their antioxidant and anti-inflammatory activity reported previously^46^. Phytosterols including Beta-sitosterol that present in N2 and NN2 possesses anti-inflammatory and antioxidant activity as reported previously^47^. Beta-sitosterol was reported to reduce MDA and increase antioxidant enzymes in liver of irradiated rats through regulation of the gene expression of peroxisome proliferator-activated receptor gamma. This action of beta sitosterol is due to its free radicals scavenging properties and antioxidant effect^48^. This effect could participate in reducing CVD risks in the present study. So, chitosan, ferulic acid and beta-sitosterol are proven to have hypolipidemic, antioxidant and anti-inflammatory effects which could reflect such actions in the studied nutraceuticals.

Plasma glucose was significantly reduced on administration of different nutraceuticals; ANN1 and AN1 were highly efficient while A showed the least effect in reducing blood sugar and IR. Ferulic acid was reported to provide a protection to beta cells of pancreas against destruction by reactive oxygen species^49^ thereby could reduce plasma glucose in the present study. Ferulic acid could also regulate hepatic GLUT2 gene expression in high fat and fructose-induced type-2 diabetic rat^50^. On the other hand, chitosan was shown previously to regulate blood sugar^51^. Beta—sitosterol was reported to improve blood sugar indirectly by the protection from pancreatic damage through its antioxidant property^52^. Nutraceuticals used in the present study were shown to improve insulin sensitivity, thereby could improve plasma glucose.

Liver and kidney functions were improved on administration of different nutraceuticals. AN1 and ANN1 were superior in improving both liver and kidney functions. Beta- sitosterol was reported to have both hepato and reno-protective effect^53^. Ferulic acid was shown to prevent hepatotoxicity through down-regulating the cytochrome P 2E1 and inhibiting toll-like receptor 4 signaling-mediated inflammation in mice^54^. A reno-protective effect of ferulic acid might be attributed to its antioxidant and antiapoptotic effect^55^. Also, chitosan was demonstrated previously to reduce fat accumulation in the liver thereby might improve liver function^56^. The improvement of liver function on administration of different nutraceuticals could be ascribed to their efficient reduction of both TG and cholesterol accumulation in the liver and anti-inflammatory and antioxidant effects seen in the present study.

Body weight gain and liver/body weight% were significantly reduced on different treatment except for liver/body weight% on administration of N2 and NN2. Chitosan can reduce body weight and enhance satiety as reported previously^51^. Also, ferulic acid was reported to reduce body weight and food intake which could be due to an effect on appetite^57^.

Histopathological liver examination showed improvement in both inflammation and fat deposition on administration of nutraceuticals, however this effect was more better on concomitant administration of atrovastatin with nutraceuticals which confirms the biochemical results.

Combination of bioactive ingredients in the present study in one nutraceutical could have a synergistic effect. nutraceuticals nanostructures prepared in the present study from chitosan, ferulic acid with or without beta-sitosterol showed promising equal and even superior effect towards CVDs risk compared to their original nutraceutical though used in half the dose. This could be attributed to the expected increased bioavailability of nutraceuticals nanoparticles. It was reported that the nano-compound is more bio-available than the normal form^10^. Chitosan nanoparticles can significantly reduce plasma viscosity and improve lipid profile of the dietary-induced dyslipidemic rats^10^. Chitosan nanoparticles can prolong contact time between active ingredient such as ferulic acid and the absorptive surface by interaction between its positive charges with the negative charge of mucin thus increasing absorption of ferulic acid and thus act as a sustained release carrier, allowing ferulic acid to improve both dyslipidemic and oxidative stress states. This was in agreement with the work of Trapani et al.^58^.

Intake of atorvastatin in adjunct to the nutraceuticals under the present study can improve bioavailability and effectiveness of atorvastatin therefore atorvastatin dose and its side effects could be reduced. This could be attributed to either chitosan effect in improving statin bioavailability by limiting its metabolism by CYP3A4 as reported by Anwar et al*.*^59^ or the direct improving effect of nutraceuticals towards the different parameters related to CVDs risks noticed in the present study.

## Conclusion

All studied nutraceuticals succeeded to improve dyslipidemia, fatty liver, plasma non-HDL-C, TC/HDL-C, oxidative stress, IR, hs-CRP, adiponectin and glucose and liver and kidney function with variable degrees in metabolic syndrome rat model. Nutraceuticals composed of chitosan and ferulic acid were more efficient in reducing CVDs risks than that containing chitosan, ferulic acid and beta-sitosterol. Nutraceuticals nanoparticles had superior effect compared to their original forms. Concomitant administration of atrovastatin with nutraceuticals elicited superior effect on plasma parameters and liver histopathological changes compared to either the drug or the nutraceuticals. In addition to the present experimental study, clinical researches are needed to assign the optimum dose from both nutraceuticals and drug that could elicit a profound effect.

## Materials and methods

### Materials

Sodium tripolyphosphate pentabasic was obtained from Sigma Aldrich, USA; CAS 7758-29-4. Dimethyl sulfoxide and acetic anhydride were purchased from Fluka, Germany. Polyethylene glycol monomethyl ether was obtained from Fluka, Germany CAS: 9004-74-4.

Atorvastatin (Lipona®) was obtained from Sedico for pharmaceutical industry, Egypt. All other chemicals and materials were of high grade.

### Animals

Male Sprague–Dawley rats were purchased from animal house of the National Research Centre, Giza. They were left for a week as an adaptation period while feeding balanced diet. The rats were housed individually in stainless steel cages with 12 h light–dark cycle. The animals had free access to drinking water and given food *adlibitum*. After adaptation period 88 rats with body weight ranges from 90 to 110 g were used. Experimental protocol was approved by ethics committee of the National Research Centre, Cairo, Egypt (approval number 19/175). All methods were carried out in accordance with the guidelines and regulations of the National Institute of Health Guide for Care and Use of Laboratory Animals (NIH No. 85: 23 revised 1985). All the methods are reported in accordance of ARRIVE guidelines.

### Methods

#### Preparation of nutraceuticals’ nanostructures:

##### Preparation of chitosan nano-particles

Chitosan was dissolved in 1% acetic acid solution at 60 °C allowed by filtration to remove insoluble impurities. Once dissolved, the chitosan solution was diluted with distilled water to produce chitosan solution of 0.5% (W/V) concentration with 6.0 pH. Sodium tripolyphosphate (TPP) was dissolved in distilled water at the concentration of 0.5% (W/V). Then TPP solution was poured drop wise to the chitosan solution under magnetic stirring at 1000 rpm using stirring bar. Then the mixture was stirred for additional 15 min. The formation of chitosan-TPP nanoparticles started spontaneously via the TPP initiated ionic gelation mechanism. The nanoparticles were formed at selected chitosan to TPP weight ratio of 5:1 at temperature of 25 °C. The nanoparticles were separated by centrifugation at 9000 rpm for 45 min. Then the supernatants were discarded. Nanoparticles were extensively rinsed with distilled water and dried at room temperature.

##### Preparation of monopolyethylene glycol (MPEG) aldehyde

MPEG-aldehyde was prepared by oxidation of PEG with dimethyl sulfoxide (DMSO)/Acetic anhydride^60,61^. After PEG completely dissolved in 30 ml DMSO; 10.2 ml acetic anhydride was added into the mixture under a nitrogen atmosphere. The molar ratio of acetic anhydride to OH of PEG was (20:1). The mixture was stirred for 9 h at room temperature under a nitrogen atmosphere. The reaction mixture was then poured into 400 ml of diethyl ether. The precipitate was filtered with a Whatman filter paper (No. 2) and then re-dissolved in small amount of methylene chloride followed by re-precipitation with diethyl ether. After air drying white powder of 8.2 g MPEG aldehyde was obtained.

##### Formation of the nutraceuticals’ nanostructures (NN1, NN2)

A mixture of 0.5 g Chitosan nanoparticles, 7.6 g MPEG aldehyde, 0.02 g Ferulic acid with or without 0.23 g β-sitosterol in 100 ml dimethyl formamide (DMF) was heated at 50 °C under magnetic stirring for 24 h. The solution was filtered, and diethyl ether was poured slowly into the filtrate under stirring. Un-reacted MPEG aldehyde was removed by suspending the precipitated Chitosan-PEG aldehyde in a large amount of dichloromethane three times. Thereafter, the precipitate was collected by filtration and dried in air and weighed.

##### Measurement of the particle size of the nutraceuticals’ nanostructures by transmission electron microscope (TEM)

Two drops from colloidal nano-preparation solution were placed on 400 mesh copper coated by carbon film and then the grids were conducted to TEM to characterize the morphology and distribution of the preparation.

##### Fourier-transform infra-red (FT-IR) Spectrometry of the nutraceuticals’ nanostructures

This step was used to confirm the trapping of active constituent (ferulic acid or beta-sitosterol or both) by chitosan mesh. Inter-particular spaces are in nanoscale, so if ferulic acid or beta-sitosterol are trapped, then they would be broken to be in nanosize.

#### Preparation of nutraceuticals in the original form

Nutraceutical 1 (N1) was prepared by mixing chitosan with ferulic acid in the ratio of (25: 1). Nutraceutical 2 (N2) was consisted of (chitosan + ferulic acid + β-sitosterol) in the ratio of (25: 1: 11.5). Nutraceutical nanoparticles 1 (NN1) and 2 (NN2) were prepared as in the previous step. It is to be noticed that chitosan was used previously as 250, 500 and 1000 mg/kg rat body weight^62^. Ferulic acid dose was reported to be 10, 20 and 40 mg/kg rat body weight^63^. Sitosterol dose for human was demonstrated to be 2- 3 g/day^64^; which is equivalent to about 180- 270 mg/kg rat body weight according to Paget and Barnes^65^ i.e. about 230 mg/ kg on average. So, it was decided to supplement rats by chitosan, ferulic acid and beta-sitosterol as 500, 20 and 230 mg/kg rat body weight, respectively from each original nutraceuticals and half these doses from the nutraceuticals’ nanostructures in the present study.

#### Preparation of nutraceuticals for administration by rats

Tween 20 (polyoxy ethylene sorbitan monolaurate) was used as emulsifying agent for both nutraceutical 1 and 2 in original and nanostructures to facilitate dosing to rats. Tween 20 was used as 10% of the nutraceuticals that were emulsified in water. Control rats were given only the vehicle.

### Preparation of diets

Two main diets were prepared; a balanced and a high fructose-saturated fat (FF) diet. A balanced diet consisted of 12.3% casein, 10% safflower oil, 68.02% starch, 3.5% mineral mixture, 1% vitamin mixture, 5% cellulose and 0.18% methionine. Diet for induction of metabolic syndrome was prepared from 12.3% casein, 25% coconut oil, 56.77% fructose, 3.5% mineral mixture, 1% vitamin mixture, 1% cholesterol, 0.25% choline and 0.18% methionine as high fructose-high saturated fat diet (HFFD) which is similar to the work of Al-Okbi et al.^3^.

### Design of animal experiment

**Figure 3:**
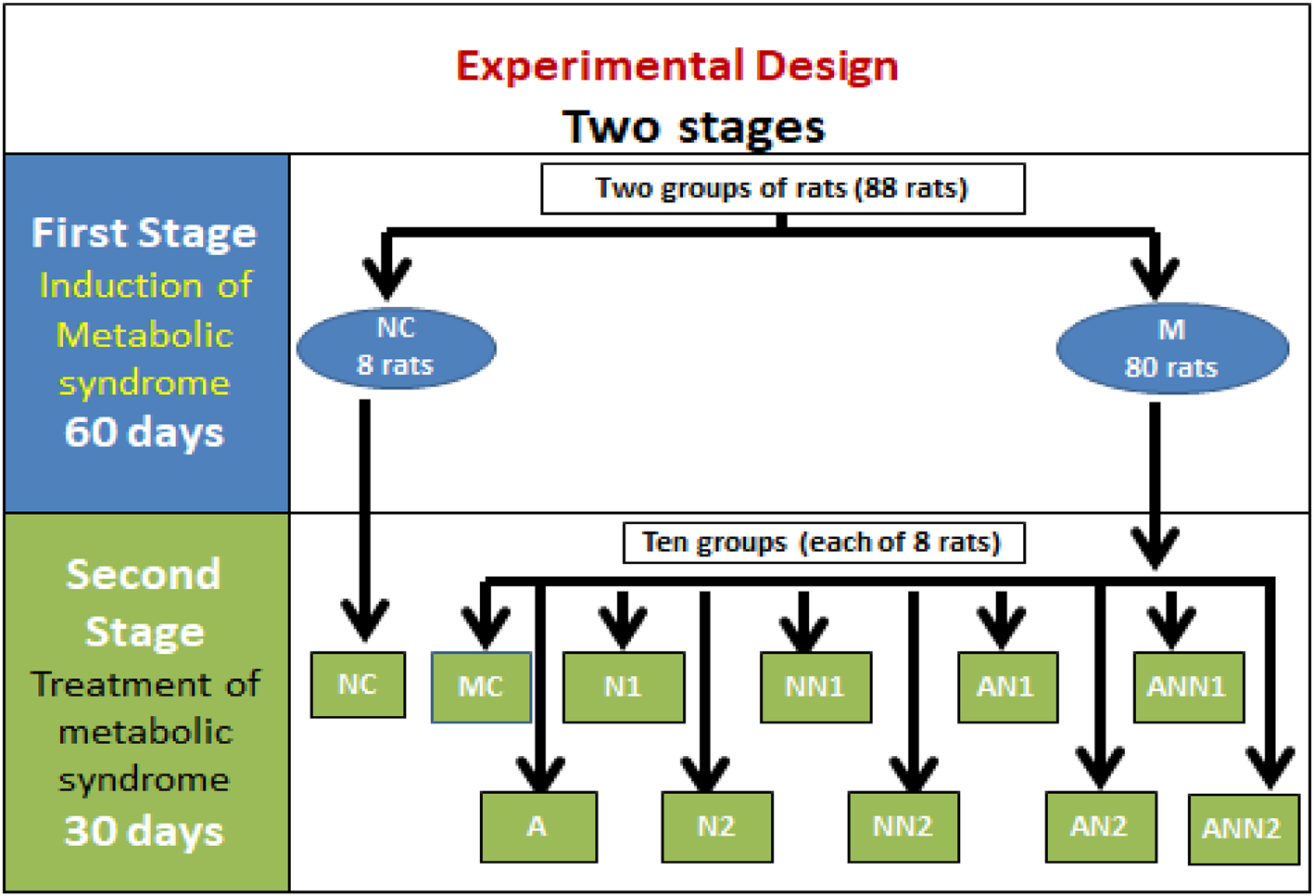
Design of the animal experiment. *M* rats with metabolic syndrome, *NC* control normal, *MC* metabolic syndrome control, *A* rats given atorvastatin drug, *N1* rats received nutraceutical 1, *NN1* rats treated with nutraceutical 1nanostructure, *N2* rats received nutraceutical 2, *NN2* rats given nutraceutical 2 nanostructure, *AN1* rats taken atorvastatin drug with nutraceutical 1, *ANN1* rats given atorvastatin drug with nutraceutical 1 nanostructure, *AN2* rats treated with atorvastatin drug with nutraceutical 2, *ANN2* rats received atorvastatin drug with nutraceutical 2 nanostructure.

The experiment was divided into 2 stages (Fig. 3). In the first stage rats were assigned to two dietary groups. The first group consists of 8 rats and served as normal control group (NC) fed on a balanced diet. The second group which was designed to induce metabolic syndrome (M) consists of eighty animals fed on HFFD. This stage was continued for 2 months. The second stage started after development of metabolic syndrome, the NC group continued on feeding balanced diet (first group), while the second group (M) was subdivided into 10 subgroups, each of 8 rats and continued feeding HFFD; a metabolic syndrome control subgroup without any treatments (MC), a subgroup administered atorvastatin as 10 mg/kg rat body weight according to Lau et al*.*^39^, a subgroup given N1as 520 mg/kg, a subgroup treated by NN1 as 260 mg/kg, a subgroup administered N2 as 750 mg/kg, a subgroup treated by NN2 as 375 mg/kg, a subgroup given N1 as 520 mg/kg rat body weight with atorvastatin as 10 mg/kg, a subgroup treated by NN1 as 260 mg/kg with the same atorvastatin dose, a subgroup administered N2 as 750 mg/kg rat body weight with the same previous dose of atorvastatin, a subgroup given NN 2 as 375 mg/kg with the same previous dose of atorvastatin. All nutraceuticals and atorvastatin doses were given as daily oral doses with gastric tubes. Body weight and food intake were recorded once weekly. At the end of experiments body weight gain (BWG), total food intake (TFI) and food efficiency ratio (FER) (Body Weight Gain /Total food intake) were calculated. Rats were overnight fasted (14 h) and anesthetized by intraperitoneal injection of pentobarbital (50 mg/kg rat body weight). Blood samples were withdrawn from rats and received into heparinized tubes. Plasma was separated after centrifugation at 3000 rpm for 15 min. Plasma glucose and lipid profile represented by total cholesterol (TC), HDL-cholesterol (HDL-C), LDL-cholesterol (LDL-C) and triglycerides (TG) were determined by colorimetric methods^66–70^. Non HDL-C (Non HDL-C = TC – HDL-C) and TC /HDL-C were calculated as risk factors for CVDs. Plasma total antioxidant capacity (TAC) and malondialdehyde (MDA) were assessed to reflect antioxidant state and lipid peroxidation, respectively^71,72^. TAC determination based on the reaction of antioxidants present in the sample with a defined amount of exogenously provided hydrogen peroxide that produces reduction in hydrogen peroxide concentration. The residual hydrogen peroxide was determined colorimetrically by an enzymatic reaction where 3,5-dichloro-2-hydroxybenzenesulphonate is converted by hydrogen peroxide to a colored product. For MDA assessment, thiobarbituric acid reacts with MDA in an acidic medium at temperature of 95 ºC for 30 min to form thiobarbituric acid reactive product. The absorbance of the resultant pink product is measured at 534 nm. Plasma alanine aminotransferase (ALT), and aspartate aminotransferase (AST) activities were determined colorimetric method according to Reitman and Frankel^73^ as biomarkers of liver function. Kidney function was assessed through estimation of plasma urea^74^ and creatinine by colorimetric techniques^75^. Plasma insulin activities were estimated using the enzyme-linked immune-sorbent assay (ELISA) using kit manufactured by Glory Science Co., Ltd, USA. Plasma high sensitivity-C reactive protein (hs-CRP) and adiponectin were assessed as inflammatory biomarkers. Adiponectin was estimated using Rat Adiponectin ELISA kit, Catalogue No.: E90605Ra, manufactured by Wuhan EIAab Science Co., China). The plasma levels of hs-CRP were determined using the ELISA kits; purchased from Glory Science Co., Ltd, USA. Insulin resistance (IR) was calculated according to homeostatic model assessment of insulin resistance (HOMA-IR) [IR = fasting plasma glucose (mg/dl) X fasting plasma insulin activity (µIU/ml) /405]^76^. Rats were dissected after euthanasia by cervical dislocation under anesthesia; livers were separated and weighed for calculation of the percentage of liver/body weight. Parts of liver were used for determination of liver lipids. Liver lipids were extracted using dichloromethane-methanol mixture according to Cequiez-Sanchez et al*.*^77^. Hepatic TG and TC were determined as previously^67,70^. Other liver specimens were kept in 10% formalin for histopathological examination. Paraffin sections were stained with hematoxylin–eosin (H&E) and images were captured using camera attached to the microscope and processed using Adobe Photoshop version 8.0.

### Statistical analysis

Statistical analysis was carried out using SPSS version 22. All data are presented as mean ± S.E. One-way ANOVA (with LSD as post hoc test) were utilized to assess the significance of differences among the studied groups at p ≤ 0.05 in the second stage while student’ s t-test was used in the first stage. Pearson correlation test was also applied to study the correlation between different biochemical parameters.

## Supplementary Information

Supplementary Information.

## Data Availability

All data generated or analyzed during this study are included in this published article.
