# Supplementary material for: Management of metabolic syndrome by nutraceuticals prepared from chitosan and ferulic acid with or without beta-sitosterol and their nanoforms
Source: Sci Rep. 2023 Jul 27;13:12176. doi: 10.1038/s41598-023-38837-9 (PMC10374579; doi:10.1038/s41598-023-38837-9)
Supplement: Supplementary file 1 — Supplementary Information. [file 41598_2023_38837_MOESM1_ESM.pdf]

Supplementary tables

Table (1): FT-IR peaks position and intensity of Chitosan

No.	Position	Intensity
1	3431.71	27.8202
2	2922.59	36.4032
3	2870.52	37.2548
4	1655.59	45.1007
5	1601.59	47.3692
6	1422.24	51.869
7	1379.82	50.5166
8	1320.04	54.34
9	1255.43	60.4312
10	1156.12	51.4823
11	1032.69	50.3304
12	897.701	64.4457
13	563.112	63.5834
14	445.476	71.5945
15	415.585	69.9451

Table (2): FT-IR peaks position and intensity of ferulic acid

No	Position	Intensity
1	3427.85	58.4577
2	2939.95	34.328
3	1639.2	85.1584
4	1461.78	63.3565
5	1375.96	66.9863
6	1243.86	88.0212
7	1188.9	88.189
8	1132.97	86.2766
9	1050.05	68.6943
10	956.52	85.8798
11	837.919	89.2023
12	801.278	87.2083
13	568.898	82.9745
14	489.831	84.1725
15	457.047	84.2922
16	412.692	90.2694

Table (3): FT-IR peaks position and intensity of β-sitosterol

No	Position	Intensity
1	3442.31	6.15534
2	2934.16	8.71692
3	2098.17	70.9726
4	1966.07	72.5725
5	1715.62	36.0173
6	1641.13	18.5229
7	1518.67	31.7074
8	1466.6	20.3642
9	1377.89	23.7938
10	1353.78	24.4709
11	1283.39	32.5668
12	1252.54	31.0243
13	1102.12	4.95832
14	987.375	74.0764
15	951.098	32.654
16	838.883	57.6288
17	806.099	70.1431
18	778.136	77.7093
19	566.969	43.0324

Table (4): FT-IR peaks position and intensity of Chitosan/Ferulic acid

No	Position	Intensity
1	3433.64	14.5982
2	2910.06	21.615
3	2834.85	21.3871
4	2662.25	24.8395
5	2588	23.7529
6	1826	81.0175
7	1680.66	7.59661
8	1606.41	5.76698
9	1511.92	7.64936
10	1423.21	9.05802
11	1272.79	3.27993
12	1172.51	3.49947
13	1030.77	15.7312
14	937.235	9.88745
15	851.418	22.3864
16	802.242	25.7434
17	749.209	46.7596
18	685.57	46.1658
19	570.826	15.0868
20	515.865	23.3254

Table (5): FT-IR peaks position and intensity of Chitosan/Ferulic acid / β-sitosterol.

No	Position	Intensity
1	3427.85	33.8855
2	2958.27	11.091
3	2937.06	10.027
4	2867.63	18.0317
5	1665.23	70.3621
6	1635.34	70.4632
7	1464.67	34.9527
8	1379.82	36.1795
9	1330.64	64.8225
10	1280.5	76.898
11	1243.86	74.5473
12	1191.79	75.7452
13	1133.94	72.222
14	1108.87	71.3041
15	1047.16	39.4286
16	987.375	81.3949
17	956.52	69.0027
18	925.664	86.2739
19	880.345	89.4123
20	839.847	80.6939
21	801.278	76.2458
22	736.674	84.4643
23	624.823	76.7586
24	592.039	76.2652
25	501.401	84.9849
26	451.261	89.4186

Table: 6. Nutritional parameters of rats fed on balanced and HFFD for two months (First stage).

Parameter	Groups	
	NC	M
IBW (g)	100 ± 5.3	100 ± 1.5
FBW(g)	207.5 ± 4.4	255* ± 1
BWG(g)	107.5 ± 2.4	155* ± 1.1
TFI(g)	749 ± 37.7	728 ± 8.8
Food intake (g/day)	12.5 ± 0.63	12.1 ± 0.15
FER	0.144 ± 0.005	0.213*±0.003

Values are expressed as means± SE (n=8 in NC group, while in M group n= 80), IBW: initial body weight, FBW: final body weight, BWG: body weight gain, TFI: total food intake, FER: food efficiency ratio, M: Rats with metabolic syndrome, NC: Normal control group.*: Significant difference from NC where p< 0.05 (Student′ s t-test).

Table 7. Pearson's correlation coefficients of the different measured biochemical parameters.

	Glucose	TC	HDL-C	Non HDL-C	LDL-C	TG	ALT	AST	Cr.	urea	TAC	MDA	Insulin	Adipo	hs-CRP	IR
Glucose	1															
TC	0.574**	1														
HDL-C	-0.295**	-0.632**	1													
Non HDL-C	0.570**	0.999**	-0.666**	1												
LDL-C	0.556**	0.998**	-0.67**	0.99**	1											
TG	0.702**	0.841**	-0.490**	0.838**	0.82**	1										
ALT	0.793**	0.555*	-0.267**	0.550**	0.539**	0.650**	1									
AST	0.745**	0.769**	-0.476**	0.767**	0.759**	0.773**	0.798**	1								
Cr.	0.712**	0.599**	-0.385**	0.598**	0.591**	0.635**	0.682**	0.731**	1							
Urea	0.610**	0.759**	-0.481**	0.758**	0.752**	0.728**	0.656**	0.870**	0.608**	1						
TAC	-0.585**	-0.758**	0.414**	-0.753**	-0.748**	-0.705**	-0.664**	-0.818**	-0.523**	-761**	1					
MDA	0.743**	0.832**	-0.552**	0.833**	0.827**	0.795**	0.688**	0.840**	0.655**	0.821**	-0.730**	1				
Insulin	0.420**	0.677**	-0.426**	0.677**	0.676**	0.565**	0.534**	0.667**	0.433**	0.620**	-0.647**	-0.633**	1			
Adipo	-0.698**	-0.899**	0.577**	-0.899**	-0.896**	-0.795**	-0.658**	-0.839**	-0.695**	-0.809**	0.731**	-0.900**	-0.644**	1		
hs-CRP	0.729**	0.535**	-0.376**	0.537**	0.524**	0.672**	0.652**	0.652**	0.606**	0.642**	-0.476**	0.694**	0.332**	-0.612**	1	
IR	0.891**	0.719**	-0.408**	0.716**	0.707**	0.753**	0.803**	0.83**	0.704**	0.718**	-0.698**	0.817**	0.781**	-0.789**	0.66**	1

*: Significant at $p < 0.05$, **: Significant at $p < 0.01$, -: Means negative correlation, no sign: Means positive correlation

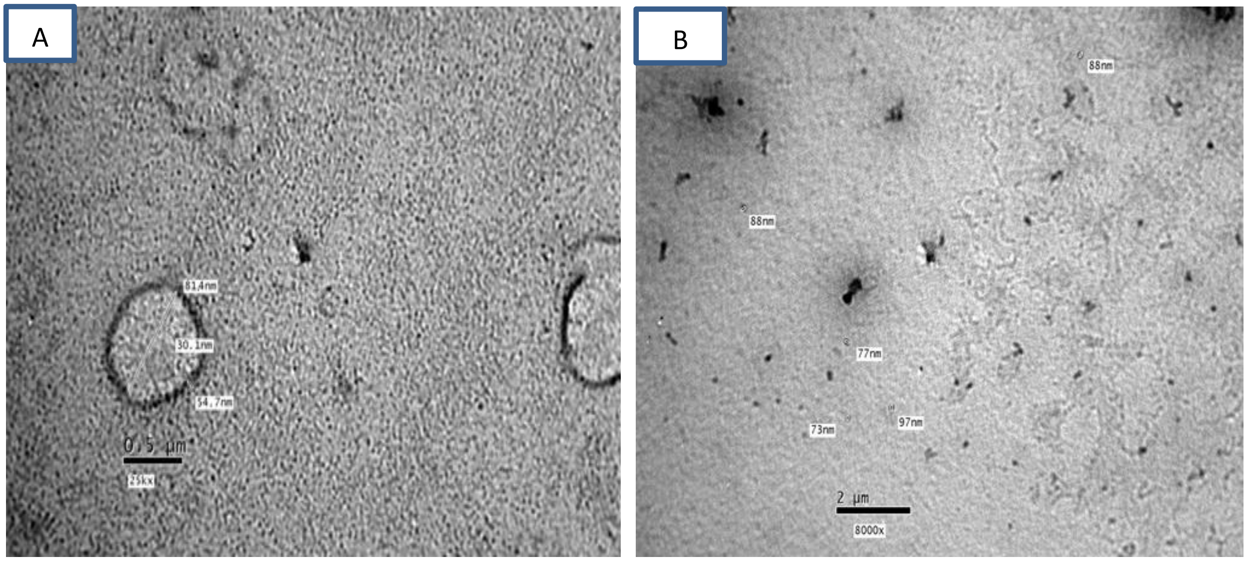

Figure 1: Particle size by Transmission electron microscopy (TEM). A: TEM of Chitosan/ Ferulic acid, B: TEM of Chitosan/Ferulic acid / β-sitosterol
